# Potential habitat distribution of Himalayan red panda and their connectivity in Sakteng Wildlife Sanctuary, Bhutan

**DOI:** 10.1002/ece3.6874

**Published:** 2020-10-28

**Authors:** Sonam Tobgay, Nattapon Mahavik

**Affiliations:** ^1^ Sakteng Wildlife Sanctuary Department of Forest and Park Service Tashigang Bhutan; ^2^ Department of Natural Resources and Environment Naresuan University Phitsanulok Thailand

**Keywords:** Circuitscape, habitat and connectivity model, Himalayan red panda, linkage mapper, maxent, Sakteng Wildlife Sanctuary

## Abstract

Survival of endangered Himalayan red panda is threatened by ever‐growing anthropogenic activities leading to an unprecedented rate of habitat degradation and loss. However, limited studies have been conducted in the context of the spatial distribution of habitats and habitat connectivity for the species in the landscape of Sakteng Wildlife Sanctuary (SWS). Lack of such information remains a challenge while implementing effective and holistic conservation initiatives. Therefore, this study identifies the distribution of potential habitats and their connectivity using maxent and linkage mapper, respectively. Precipitation‐related predictor variables exhibited a significant influence on the prediction of habitat distribution. The model predicted 27.7% of the SWS as a potential habitat (fundamental niche). More than 75% of the predicted habitats fall outside the existing core zones where anthropogenic disturbance is relatively high, indicating the need to reassess existing management options. In SWS, 15 core habitats (CH) are predicted which are connected by a least‐cost corridor (length µ = 2.91 km) with several pinch points in it. Centrally located CH5 and CH11 are identified as the most important habitat in maintaining overall connectivity within SWS. However, CH located in the peripheries could be equally important in facilitating the transboundary movement of the species. Overall, SWS can play a critical role as a connecting link between the larger landscape of Bhutan and the adjacent Indian state of Arunachal Pradesh in the conservation of Himalayan red panda that exhibits narrow dispersal with special habitat needs. Based on our findings, we recommend initiating GPS/satellite telemetry of the species to enable SWS to understand the precise interaction of Himalayan red panda to widespread herder communities, livestock, and free‐roaming dogs dwelling in the same landscape. It will also help to evaluate the functionality of the predicted habitats, linkages, and feasibility of transboundary conservation initiatives.

## Graphical abstract

This article is about the application of habitat and connectivity model to identify the potential habitat and linkages between the predicted habitats with regard to the ecological need of Himalayan red panda. This study was conducted in Sakteng Wildlife Sanctuary in Bhutan. According to the model, Himalayan red panda in the study area is likely to experience a high frequency of anthropogenic disturbances. However, these predicted habitats are found to be an important connecting link between the larger landscape of Bhutan and the Indian state of Arunachal Pradesh facilitating the transboundary movement of the species.

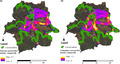

## 1 INTRODUCTION

The dynamics of habitat, connectivity, and their influence on survival of the species is an inevitable component of wildlife ecology (Morrison et al., 2012). Habitat is an area in the landscape that can support a viable population of the species. It fulfills the fundamental needs of an individual or the population to reproduce, occupy, protect, interact, and survive by providing food, shelter, water, and climatic or environmental conditions in favor of the species (Morrison et al., 2012). However, a healthy viable population of wildlife is dependent on a mosaic of heterogeneous habitat across the landscape (Grebner et al., 2013) with good connectivity (Fahrig & Merriam, 1994).

Habitat connectivity is the extent to which species can move between the fragmented landscape (Taylor et al., 1993). Connectivity is critical for facilitating effective dispersal of the species across the landscape, uninterrupted seasonal migration, population persistence, and range expansion and maintains prey–predator dynamics (Cross et al., 2013; Kareiva & Wennergren, 1995; Stephens & Krebs, 1986; Taylor et al., 1993). Connectedness helps in the maintenance of ecosystem functionality and biodiversity in the landscape.

Eastern Himalaya is home to diverse wildlife including several globally threatened species. It extends from Koshi Valley in Central Nepal through northeast India, Bhutan, the southeast Tibet to northwest Yunnan in China, and northern Myanmar (Chettri et al., 2018; Sharma et al., 2009). Mountainous topography with varying vertical climatic zonation of microhabitats and restricted species distribution makes the region known for many endemic species. The Himalayan red panda *Ailurus fulgens* is one of the endangered species endemic to Himalaya (Glatston et al., 2015; Hu et al., 2020). They are distributed across the temperate region from western Nepal to southern Tibet till Yarlung Tsangpo via northeast India (Darjeeling, Sikkim, and Arunachal Pradesh) and Bhutan (Hu et al., 2020).

Red panda belongs to a family of Ailuridae (Duszynski et al., 2018) which feeds on bamboo despite being categorized in the order of carnivora. The young leaves and shoots of bamboo comprise of the primary diet, often supplemented with fruits, mushrooms, succulents, roots, and acorns (Yonzon & Hunter, 1991). It is mostly arboreal and has specialized habitat niche requirements related to forest types, elevation, availability of fallen logs and stumps, proximity to water sources, and disturbances (Bista et al., 2017; Wei et al., 1999; Yonzon & Hunter, 1991). They prefer fir‐dominated mixed deciduous–coniferous forest of the temperate zone with profuse bamboo undergrowth (Bista et al., 2019; Glatston et al., 2015).

Wild population of Himalayan red panda is estimated to be very small (Glatston et al., 2015; Wang et al., 2008) which are likely to further decrease due to increasing anthropogenic and climate change‐induced threats. Subsequently, it will result in very low genetic diversity, high linkage disequilibrium, and high genetic load that is detrimental to species population growth (Glatston et al., 2015; Hu et al., 2020). Human population residing within the stretch of the Himalayan red panda habitat across eastern Himalaya are some of the poorest people whose dependence on natural resources is immense (Sandhu & Sandhu, 2015). This, in turn, elicits disturbances that are undesirable to the species (Acharya et al., 2018). Although it is legally protected throughout the range countries and included in Appendix I of CITES (Glatston et al., 2015), habitat loss, degradation, fragmentation, mass flowering of bamboo, climate change, resource competition, attack by dogs, and increased incidences of poaching and illicit trade of the species are threatening its survival (Bista et al., 2017; Dendup et al., 2016; Dorji et al., 2012; Glatston et al., 2015; Wei et al., 1999; Yonzon & Hunter, 1991).

Generally, Himalayan red panda in Bhutan occurs within the elevation range of 2,400 to 3,700 m in cool broadleaf to fir forest with good bamboo undergrowth preferably near water sources (Dorji et al., 2012). In 2011, Himalayan red panda was reported to be present only in 13 districts (Dorji et al., 2012); however, recently their distribution in 17 out of 20 districts was confirmed (NCD, 2019). Out of 10 protected areas (PAs) and 9 biological corridors (BCs), the presence of the species is documented from 7 PAs and 8 BCs. Twenty‐one percent (or 8,062.74 km^2^) of the Bhutan's geographical area is predicted as potential habitat for Himalayan red panda, out of which 62% are within the network of PAs. However, only 21.4% were predicted to be moderately to highly suitable (Dorji, 2011). Although Himalayan red panda is legally protected as Schedule I species (RGOB, 1995) and the majority of potential habitats falls within the network of PAs, it still experiences threats from communities residing in the same elevation range in and outside the PAs (Dorji, 2011). The threats include timber and fuelwood extraction, construction of roads, growth in tourism sector, people's dependence on natural resource, extensive livestock grazing, inadvertent poaching, and predation by dogs (Dendup et al., 2016, 2020; Dorjee, 2009; Dorji et al., 2012).

**Figure 1:**
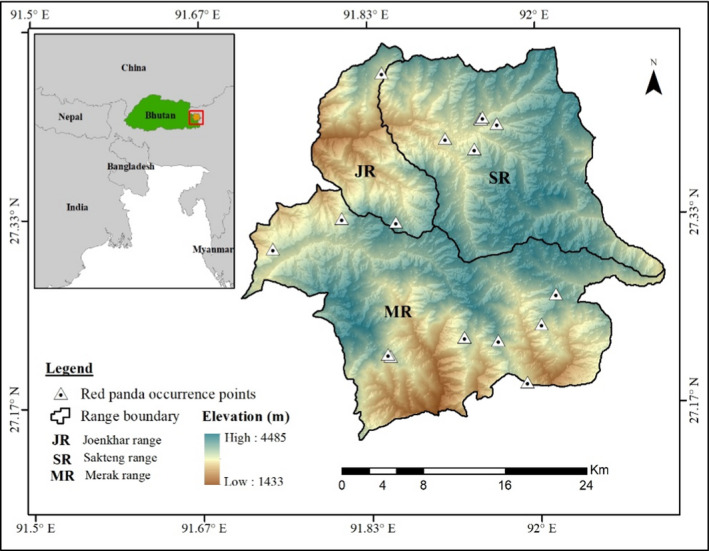
Map showing the location of Sakteng Wildlife Sanctuary (study area) with distribution of Himalayan red panda occurrence points

Sakteng Wildlife Sanctuary (hereafter SWS) is one of the ten PAs in Bhutan (Figure 1). It is divided into three ranges, Merak, Sakteng, and Joenkhar, and managed under different zones based on their prime functions. The core zones are designated for strict conservation where activities other than conservation works and research are prohibited. The buffer zones are transition zone between the area within and outside SWS which functions as the cushion against potential impact from outside. In between the two zones lies the multiple‐use zone designated for multiple use with few restrictions (WWF & SWS, 2011). The timber extraction site is a part of multiple‐use zones that are designated for extraction of timber resource to meet the growing demand of timber for local use (SWS, 2019). Except for the core, other zones are likely to experience a high frequency of anthropogenic disturbances.

Himalayan red panda occurs in the fir and mixed conifer forest of this sanctuary. However, SWS is also a home to indigenous seminomadic community known as Brokpa, group of yak and cattle herder. More than 85% of the inhabitants’ livelihood is dependent on livestock rearing (SWS, 2019). Their winter rangeland overlaps with the primary habitat of the Himalayan red panda.

Recent studies in Bhutan predicted increased in temperature and rainfall by 3.2°C and 30%, respectively, toward the end of the century (NCHM, 2019) which in turn increases the vulnerability of habitats to climate change. Therefore, climate change, resources demand by huge livestock population, and increasing timber needs by the inhabitant exert intense pressure on forests in SWS which are important habitat for Himalayan red panda.

Even though Himalayan red panda is identified as the priority species for conservation, information regarding the spatial distribution of the potential habitat and their connectivity in SWS is very limited. Lack of reliable information remains a challenge while implementing effective and holistic conservation initiatives. Hence, this study will identify potential habitat distribution and their connectivity for Himalayan red panda within the landscape of SWS to ensure the long‐term survival of the species.

## 2 MATERIALS AND METHODS

### 2.1 Occurrence point and predictor variables

Himalayan red panda occurrence points were collected during national tiger survey (October 2014–May 2015), biodiversity survey (2015–2016), musk deer camera trapping exercise (June–November 2017), sustainable forest management plan survey (November 2017–March 2018), and regular field patrolling. Himalayan red panda was identified based on photographs captured by camera traps, scats, and their feeding characteristics. Occurrence coordinates were recorded using handheld GPS. Systematic national tiger survey was conducted by the installation of 16 pairs of camera traps in every 5 km × 5 km grid across the SWS. Biodiversity survey in 108 circular plots (12.62 m radius) followed the stratified random sampling, and the musk deer survey used an opportunistic survey by installing 10 numbers of camera traps. Sustainable forest management plan adopted a systematic survey that was confined within potential resource extraction sites. Data collected during patrolling did not follow any defined survey method; however, extensive patrolling is regularly conducted within SWS irrespective of seasons. After screening, 18 georeferenced occurrence points were selected for this study.

Nineteen bioclimatic variables with 30 arc‐second spatial resolution (Hijmans et al., 2005) were downloaded from www.worldclim.org/bioclim . It consists of annual trends (mean annual precipitation and temperature), seasonality (annual range in precipitation and temperature), and extreme or limiting environmental factors (temperature of the coldest and warmest month, and precipitation of the wet and dry quarters). They are represented by the average climate of the year 1950–2000 (Hijmans et al., 2005). Slope, aspect, and elevation layers were derived from digital elevation model.

### 2.2 Selection of predictors and modeling process

Multicollinearity was minimized by selecting only variables with Pearson's correlation (*r*) < 0.7 (Zhu & Peterson, 2017). Background sample points were restricted within the 4km buffer of actual points of species occurrence to reduce the sampling bias. Since home range size of Himalayan red panda is predicted to be < 4 km^2^(Johnson et al., 1988), area within 4km buffer seems reasonable for approximating the assumptions of background selection by excluding large areas that the species does not reside because of dispersal limitations or biotic interactions. Model was fine‐tuned with help of ENMeval package in R with given settings: method = "randomkfold" (where kfolds = 5), RMvalues = seq (0.5,4,0.5), and fc = c ("L," "LQ," "H," "LQH"). Model with lower corrected Akaike's criteria (AIC_c_) values with less overfitting was selected as the best‐fit model. AICc accounts balance between the goodness of fit and number of the model parameters enabling selection of model with optimal complexity (Warren & Seifert, 2011). Model was trained and tested with 80% and 20% of the occurrence points, respectively. Prevalence‐independent maximum true skill statistics (TSS) value was selected as a decision threshold to distinguish between suitable and unsuitable habitats (Somodi et al., 2017). The pixels with values equal to or higher than the threshold are considered as suitable habitats, yielding a binary prediction map. Jackknife test determined relative importance of individual predictors. The model was executed in maxent version 3.4.1 using dismo package in R v3.4.0.

Model performance was assessed based on the area under curve (AUC) of the receiver operating characteristic (ROC) plot. It measures the distinguishing capability of the classification model where ROC represents the probability curve and AUC as the measure of separability. AUC‐ROC tells how much the model is capable of discriminating between the classes. The AUC threshold ranges from 0 to 1 where value higher than 0.5 is considered as a good model with better discriminatory capability (Jiménez‐Valverde, 2012; Phillips et al., 2006). Further, Kappa statistics were also used to assess the performance of the model. The value of the Kappa statistic ranges from minus (−) 1 to plus (+) 1, where values close to +1 indicate better performance and values =<0 indicate a poor performance (Cohen, 1960).

The raster resistance or cost file and vector core habitat file are both extracted from a habitat suitability map produced by the maxent model. Final resistance map was formed based on inverted habitat suitability map from maxent and the resistance map produced based on expert's opinion. The inverted habitat suitability map was added with reclassified normalized land use map (FRMD, 2017) with resistance value (Table S1) to form resistance surface. It represents relative cost required to pass over gridded mapped surface representing the landscape. The following equation was used to invert the habitat suitability map (Esri, 2019):

$$\text{Float}\;({(}^{\prime \prime }\text{Raster}\;{\text{layer}}^{\prime \prime }‐\text{Highest}\;\text{cell}\;\text{value})*‐1)+\text{Lowest}\;\text{cell}\;\text{value}$$

Core habitat (hereafter CH) used in connectivity model was prepared by removing nonforested areas, settlements, and timber extractions sites from the binary habitat suitability output of maxent.

Using resistance and core habitat as input files, linkage mapper version 2.0.0 is executed to map least‐cost corridors (LCC) and least‐cost paths (LCP) between the pair of core habitats. Linkage mapper identifies the adjacent core habitats, creates a network of core habitats using distance and adjacency data, calculates cost‐weighted distance (CWD) and LCP, and generates maps of LCC between them. Later, it combines all individual corridors to from normalized composite map of corridors (Dutta et al., 2015) calculated as follows.

$$CWD_{A}+CWD_{B}‐LCD_{\mathrm{AB}}$$

where *CWD_A_* is CWD from core habitat A, *CWD_B_* is CWD from core habitat B, and *LCD_AB_* is the cost‐weighted distance accumulated moving along the LCP.

LCC identifies the swath of habitat expected to provide the best route for the movement of animal between the patches of habitats. CWD denotes the least accumulative cost required to traverse between a cell and a specified source which is equal to the resistance value of individual cells to be traversed multiplied by the cell size. The LCP is the single path generated with the minimum CWD between the core habitats (Adriaensen et al., 2003). After least‐cost corridors are mapped, pinch point and current flow centrality were determined. The pinch point represents an area within the corridor that functions as the bottleneck without much alternative route for the movement (McRae, 2012a). Even small loss of areas in identified pinch points would result in compromise of the connectivity intactness (Castilho et al., 2015). Current flow centrality helps to measure the importance of a respective linkage in maintaining the overall connectivity (McRae, 2012b). During the process, LCC acts as the surface through which current will flow between the habitats and amount of flow is dependent on resistance of individual cells within it. Pinch point mapper and centrality mapper in linkage mapper were used to determine pinch point and current flow centrality, respectively. Both use circuit theory by calling Circuitscape in linkage mapper (McRae et al., 2008). Random choice of CWD cutoff width delineates what area to include within the predicted corridor. However, due to lack of empirical data on optimum width of CWD for Himalayan red panda corridor, existing model was executed with 500 m corridor cutoff width. This cutoff figure was derived based on ca. 14% (twice the core area) of Himalayan red panda's home range size since their core area constituted of only 7.6% of the home range (Johnson et al., 1988).

Corridors are analyzed and compared based on cost‐weighted ratio metrics. The two metrics are computed by means of the ratio of CWD to the Euclidean distances (EucD) separating each pair of CH and CWD to the length of LCP. The higher ratio value for first metric indicates higher difficulties to move between the CH pairs relative to how close they are or after accounting for the EucD. Second metric describes average resistance animal has to encounter while moving along the LCP identified as the optimal or least resistance path. In both cases, optimum quality linkage will have the ratio equals to one (Dutta et al., 2015).

## 3 RESULTS

### 3.1 Model selection, performance, and influencing variables in habitat prediction

From the 32 candidate models (Table S2), the best‐fit model was assessed with the lower AICc values with higher mean test AUC. The best‐fit model has an AICc value of 443.66 (RM values = LQH3.0). It exhibited higher mean training AUC (0.79) and test AUC (0.74), meaning the selected model performs better than random (AUC < = 0.5) in predicting potential Himalayan red panda habitat distribution (Figure 1). The higher kappa (train = 0.749 and test = 0.739) also suggests the better discriminatory capability of the model.

**Figure 2:**
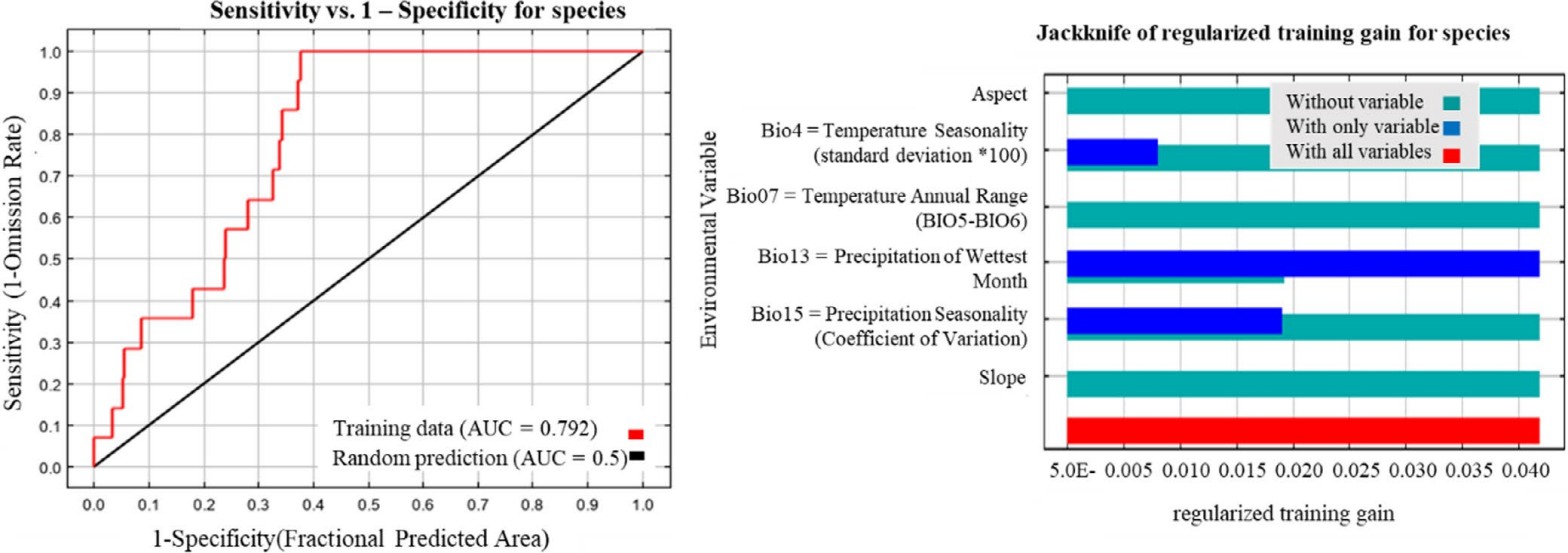
(Left) Area under the curve (AUC) plot. AUC = 0.792 suggests the good performance of the model. (Right) A jackknife test result showing the relative contribution of predictor variables in predicting the potential habitat distribution

Out of 19 bioclimatic and 3 environmental predicator variables (Table S3), only six of them had the correlation value less than the assigned threshold (*r* < 0.7) and are used for model execution. The bio13 (precipitation of wettest month) contributed 67% to model building followed by bio15 (33%) (precipitation seasonality (coefficient of variation)). While bio4 (temperature seasonality (standard deviation *100)), bio7 (temperature annual range (bio5‐bio6)), slope, and aspect did not contribute to the model (Figure 2). Overall precipitation exhibited significant influence in predicting potential habitat distribution of Himalayan red panda in SWS.

### 3.2 Distribution of potential habitat for Himalayan Red panda

The maxent model predicted that SWS is likely to have 260 km^2^ of potential habitat for Himalayan red panda. This accounts for 27.7% of the total area under SWS. Maximum habitat was predicted under the jurisdiction of Merak range (54.5%) followed by Sakteng (33.4%) and least in Joenkhar (12.2%). Although Joenkhar has small patch of potential habitat, it serves as an important link between the larger habitats of Merak and Sakteng. Based on Land Use Land Cover 2016 (FRMD, 2017), mixed conifer (60%) and fir (20.8%) comprise of major forest types within the predicted habitats.

**Figure 3:**
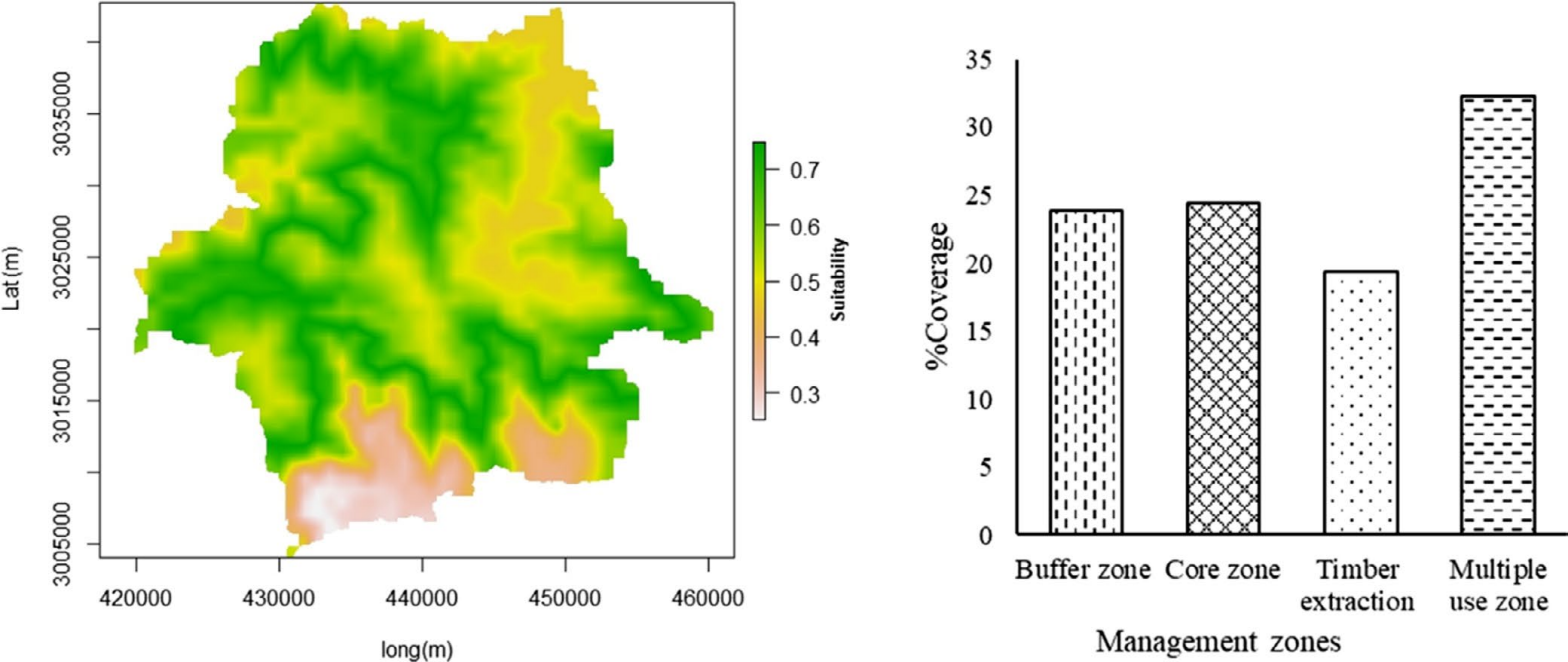
(Left) Distribution of potential habitats across SWS. Habitat suitability is illustrated in the color gradient where white and dark green indicates the lowest and highest suitability, respectively. (Right) Distribution of the potential habitats within different management zones. More than 75% of the predicted habitats fall outside the core zone where the likelihood of anthropogenic disturbance is relatively high

Out of 260 km^2^ of predicted potential habitats; only 24.4% falls within core zone and remaining 75.6% are known to occur in other zones comprising of buffer (23.9%), multiple‐use (32.3%), and timber extraction sites (19.4%) where likelihood of anthropogenic disturbance is relatively high (Figure 3).

### 3.3 Connectivity between the predicted potential habitats

In SWS, 15 core habitats (CH) with an area ranging from 0.3 to 43.3 km^2^ (µ = 11.5) were identified (Table S4). Sum of the area for 15 CH was 173.2 km^2^, which is 33.4% less than the total potential habitats predicted by maxent model. This deficit accounts for those areas predicted as potential habitats but falls within nonforested area, settlements, and timber extraction sites which were removed from CH used for connectivity analysis.

**Figure 4:**
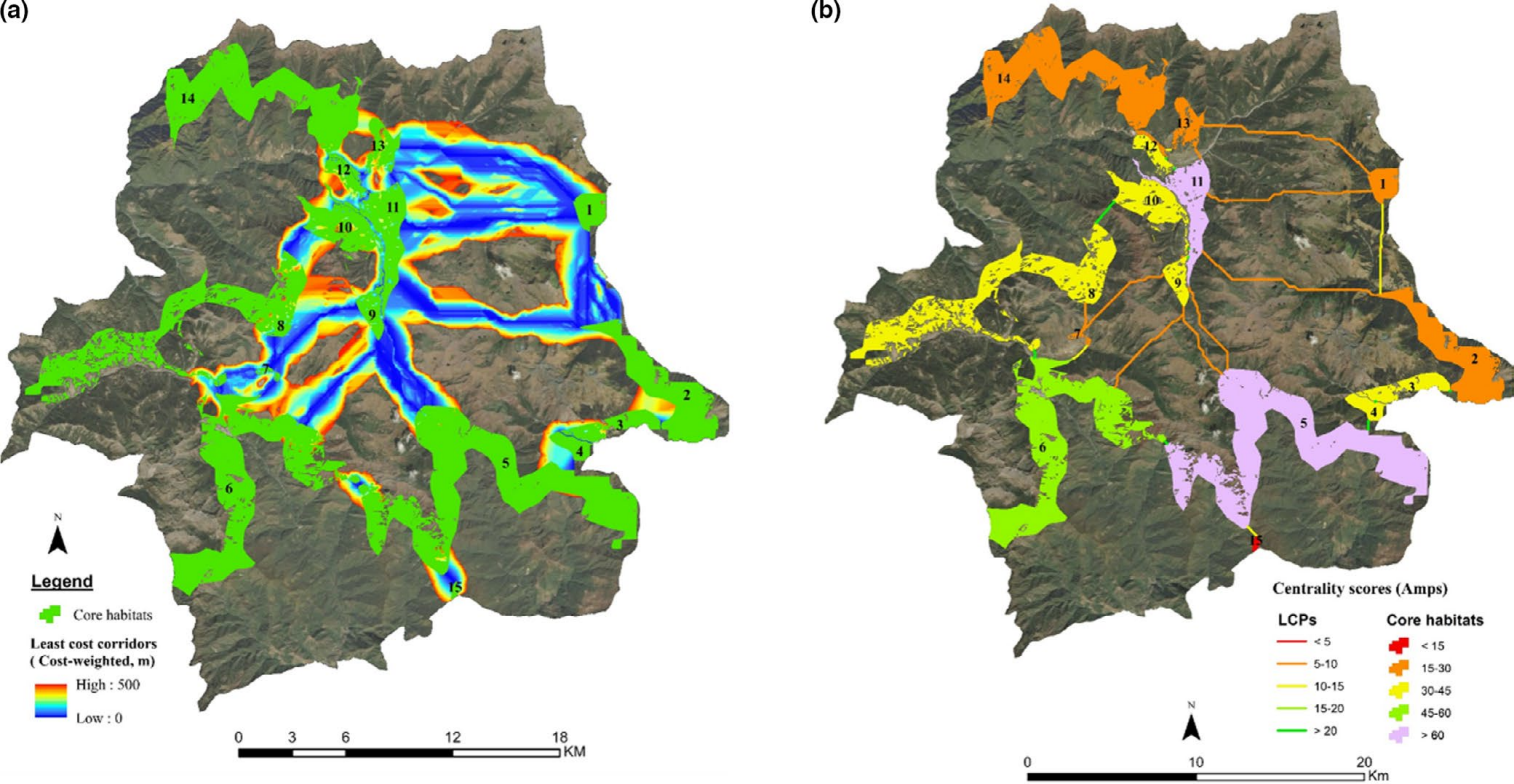
(a) Map showing the least‐cost corridors clipped at cost‐weighted distance (CWD) of 500m. CWD is illustrated in the color gradient where blue indicates the lowest cost path and red indicates the highest. (b) Centrality core habitats and least‐cost path linkages. Core habitats and linkages are color‐graded according to their centrality score. Higher scores indicate high importance

The connectivity model identified and mapped 24 active linkages across the landscape which can help to maintain connectivity between different pairs of CH (Figure 4). The EucD ranged from 0.01 to 10.45 km (µ = 2.65, σ = 3.34), CWD ranged from 0.01 to 6.1km (µ = 1.43, σ = 1.92), and LCP ranged from 0.03 to 11.24 km (µ = 2.91, σ = 3.37). The highest value of EucD (10.45 km), CWD (6.10 km), and LCP (11.24 km) was recorded for CH2‐CH11, while CH9‐CH11, CH12‐CH14, and CH10‐CH11 exhibited lowest EucD (0.01 km), CWD (0.01 km), and LCP (0.03 km).

The mean CWD:EucD and CWD:LCP was 0.67 (σ = 0.27) and 0.47 (σ = 0.15), respectively. The highest CWD:EucD (1.43) was recorded for CH3‐CH4 which indicates that the cost of species movement between CH3 and CH4 is relatively higher than other pairs of CH despite having the same EucD. The linkage between CH4 and CH5 exhibited the highest quality illustrated by lowest CWD:EucD (0.36). The highest resistance to movement along the optimal path was recorded in between CH11 and CH13 which is indicated by the highest CWD:LCP (0.98). The lowest CWD:LCP (0.26) occurred in between CH10 and CH11 demonstrating lowest resistance to movement along an optimal path (Table 1).

**Table 1:** Characteristics of 24 active linkages mapped between 15 core habitats. Linkages are sorted with decreasing current flow centrality scores to illustrate their importance in keeping the landscape connected

CH		EucD (Km)	CWD (Km)	LCP (Km)	CWD:EucD	CWD:LCP	Current flow centrality (Amps)
From	To						
5	6	0.18	0.14	0.21	0.82	0.67	38.77
4	5	0.53	0.19	0.55	0.36	0.35	38.11
11	12	0.05	0.05	0.09	0.96	0.57	32.97
3	4	0.03	0.04	0.08	1.43	0.51	32.23
10	11	0.01	0.01	0.03	0.59	0.26	26.70
12	14	0.01	0.01	0.03	0.78	0.32	24.78
2	3	0.02	0.02	0.06	1.08	0.34	24.70
9	11	0.01	0.01	0.03	0.62	0.27	24.25
6	8	0.34	0.20	0.54	0.60	0.38	23.65
8	10	1.62	0.68	1.72	0.42	0.40	23.20
13	14	0.22	0.17	0.24	0.80	0.71	14.94
5	15	0.71	0.26	0.75	0.37	0.35	14.00
6	7	1.01	0.41	1.14	0.41	0.36	13.61
1	2	5.07	2.49	5.35	0.49	0.47	11.77
5	9	4.45	2.07	4.96	0.47	0.42	9.67
5	11	5.30	2.47	5.77	0.47	0.43	8.12
7	8	1.63	0.74	1.91	0.45	0.38	7.00
7	9	5.67	3.03	6.41	0.53	0.47	6.19
1	11	9.34	5.33	10.34	0.57	0.52	5.79
1	13	10.17	5.70	11.08	0.56	0.51	5.73
11	13	0.57	0.63	0.65	1.12	0.98	5.64
9	10	0.03	0.03	0.06	0.97	0.48	5.61
6	9	6.10	3.50	6.67	0.57	0.53	5.01
2	11	10.45	6.10	11.24	0.58	0.54	4.22

Centrality scores varied among the CH and linkages. The high centrality score was recorded for CH5, CH11, CH6, and CH4, while lower scores were observed for CH15, CH7, CH1, and CH13. The highest centrality scores for CH5 (61.3) and CH11 (60.8) indicate their importance in keeping the overall Himalayan red panda habitats within SWS connected. However, area‐corrected centrality scores unveiled that CH7 (59.4) will play an extremely important role in maintaining the connectively in SWS irrespective of its size. The centrality score for linkages between CH5‐CH6 (38.77) and CH4‐CH5 (38.22) was recorded to be highest, further supporting the importance of the CH5 landscape in maintaining the overall connectivity. On the other hand, lowest centrality score was recorded for the linkage between CH2 and CH11 (4.22) indicating its minimum role in overall connectivity (Table 1).

**Figure 5:**
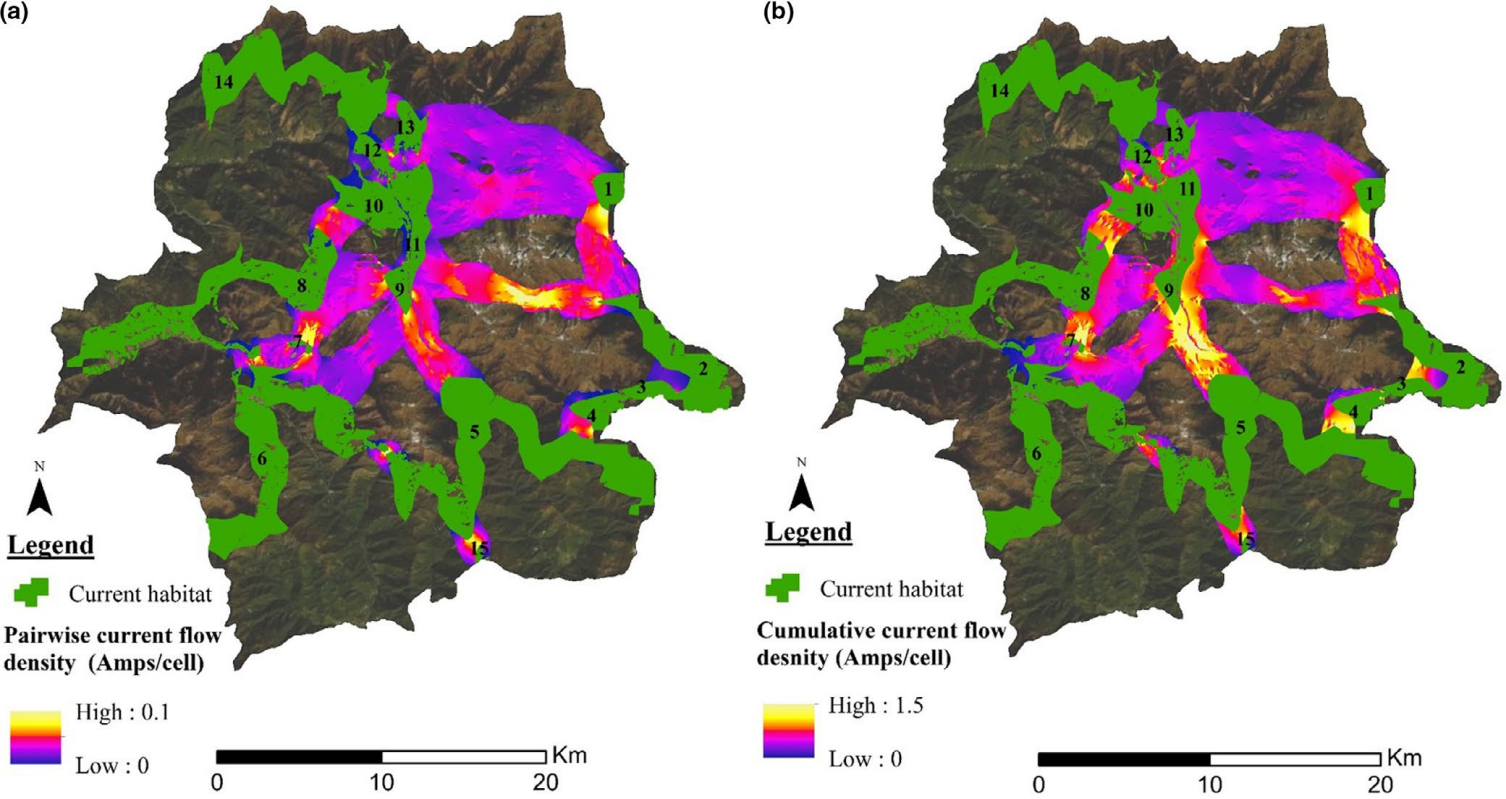
(a) Pairwise pinch points and (b) all‐pairs pinch point. Shades of yellowish indicate areas where the current flow is highly restricted representing the pinch points

The model exhibited the presence of several pinch points in the corridors being mapped. The pairwise analysis revealed occurrence of pinch points in between almost every pair of CH, while there were only a few pinch points in terms of all‐pairs analysis. Pairwise pinch points indicate constriction in movement pathways in between the two CH which is illustrated by areas with higher current flow, whereas all‐pairs analysis shows the pinch points in the connectivity illustrating part of corridors that is essential in keeping an entire network of habitat connected. The linkages between CH5‐CH9, CH7‐CH8, CH8‐CH10, CH4‐CH5, CH2‐CH3, and CH1‐CH2 have a higher all‐pairs pinch points, signifying that these are important linkages to keep the entire network of Himalayan red panda habitat connected in SWS (Figure 5).

## 4 DISCUSSION

Although model predicted 27.7% (260 km^2^) of the SWS as a potential habitat (fundamental niche), actual habitat (realized niche) is likely to be less since the correlative species distribution model predicts fundamental niche which is relatively larger than the realized niche (Polechová & Storch, 2019). Further, the distribution of species can be limited by other factors like land use, edaphic, competition, and anthropogenic disturbances that are not incorporated in the current model (Ranjitkar et al., 2014; Wang et al., 2018). Interpolated bioclimatic variables may not represent the immense variability of climate in the high mountainous region, increasing the model uncertainty. However, the predicted habitat distribution matches the anecdotally known and methodically confirmed distribution of Himalayan red panda in SWS (Dorjee, 2009). Taking into account the average density of 1 adult/4.4 km^2^ (Yonzon & Hunter, 1991) and 260 km^2^ of predicted fundamental niche, SWS is likely to support ca. 59 individuals. However, Himalayan red panda density was found to be ca. 34% (1 adult/2.9 km^2^) less in realized niche (Yonzon & Hunter, 1991). Therefore, the actual population of Himalayan red panda in SWS is expected to be approximately 39 individuals.

According to SWS (2019), Sakteng has the highest livestock population indicating relatively high competition leading to poor habitat quality in comparison with Merak and Joenkhar. Similar findings were reported in the earlier studies in Phrumsengla National Park (Dendup et al., 2016) and Langtang National Park (Yonzon & Hunter, 1991). Merak range with maximum predicted potential habitats and lower livestock population is expected to have less disturbed habitats. However, fuelwood extraction data suggest that Merak with extremely cold weather consumes fuelwood 40% and 80% higher than Sakteng and Joenkhar, respectively (SWS, 2019). Requirements of fuelwoods and timber are met from the nearby forest which suggest that habitat degradation from resource harvesting could be the issue in Merak range similar to the findings of Dorji et al. (2012) in other parts of the country.

In the mountainous topography, numerous seasonal spring flow emerges during the monsoon as a result of high orographic precipitation. Accessibility to seasonal spring flow could influence the dispersal, since earlier studies reported that Himalayan red panda has a high affinity to water accessibility (Wei & Zhang, 2011; Yonzon & Hunter, 1991). Further, precipitation is one of the important factors that regulate the growth and development of bamboo which is a primary diet of Himalayan red panda (Yonzon & Hunter, 1991). The significant contribution of precipitation‐related predictors in the current model may correspond to its influence on water accessibility and regeneration of the bamboo (Thapa et al., 2018).

Increasing research supports corridor as an important tool that can help the persistence of the species. The corridor between habitat patches plays vital role in maintaining flow of genetic exchange via interpopulation dispersal between the disconnected habitat patches (Hanski & Ovaskainen, 2000), contributing positively to demographic factors and metapopulation dynamics (Hanski, 1998). Prediction of potential linkages (least‐cost corridor) in this study provides the first account of the potential dispersal route for Himalayan red panda within the landscape of SWS. These predicted linkages represent areas with the least cost for effective dispersal of Himalayan red panda, and their importance could be explored by the management of SWS for developing a strategic conservation plan.

While movement information of Himalayan red panda is lacking in Bhutan, study in China using radio telemetry found that Himalayan red panda travels ca. 500 m per day within the home range of 3.4 km^2^ (Johnson et al., 1988). This perhaps will consume a considerable amount of time and energy to traverse between isolated habitat patches where the average length of predicted least‐cost corridors is 2.9 km. According to Johnson et al. (1988), Himalayan red panda avoids open spaces and consumes 63% of the day resting during frequent interspaced activity, each resting period lasting ≤2 hr. In SWS, some of the longest predicted least‐cost corridors (highest 11.24 km), at some points, do not pass through the vegetated area as required by the species. Thus, for Himalayan red panda which has specialized habitat need and narrow dispersal ability, identification and management of relatively small habitat patches (stepping stone) along or at proximity to the predicted corridor will facilitate their movement. However, SWS should be cautious in identification of stepping stones since insufficient size, wrong location, and poor quality stepping stones may distract species from successfully colonizing the intended larger suitable patches resulting in reduced colonization success (Kramer‐Schadt et al., 2011; Saura et al., 2014).

Though feasibility and functionality of predicted corridors are not tested in the ground, linkage quality metrics suggested that quality and significance of respective linkages varied from each other. The close inspection of the least‐cost corridor overlaid on the base map revealed that poor quality linkages occur between those core habitats isolated by rivers and unsuitable land use types. This could be attributed to the very fundamental concept that cost distance increases in proportion to the increase in resistance along the landscape.

The highest centrality score was detected in CH5 and CH11. CH11 is centrally located among the habitats in the southern region (Merak range), and CH5 represents the center of northern habitats (Sakteng range), thus indicating their importance in maintaining overall connectivity within SWS. This does not mean that CH located in the peripheries are not important for Himalayan red panda conservation. It could be equally important in facilitating the movement in the context of larger landscapes adjacent to SWS which are outside the delineated study area but are home to the Himalayan red panda. Thus, habitats located in the eastern region with relatively low centrality scores (CH1, CH2, and CH3) can play an equally important role as connecting link to enable transboundary movement of the species between Indian state of Arunachal Pradesh (east) and SWS (west). The need for transboundary landscape connectivity in this region was also recommended in earlier studies (Dorji, 2011; Thapa et al., 2018).

Pinch points were observed in all most all pairs of CH, suggesting that predicted linkages in SWS possess some kind of bottleneck in the movement of the Himalayan red panda. This can be a critical section of the linkage for maintenance of a network of connectivity. Such pinch points could be the result of one or a combination of several factors that must be evaluated via a detailed field survey. Understanding the detailed cause of pinch points and exploring potential mitigation and restoration measures will help in improving the existing network of connectivity (Dutta et al., 2015). With a visual inspection over the base map, most of the pinch points are caused by natural features though the actual ground survey might reveal otherwise. However, there is a pending proposal for the construction of hydropower plant in the Gamri River that flows through the landscape of Sakteng and Joenkhar ranges. Such man‐made infrastructures could result in collateral damage to the network of habitat connectivity, leading to an increasing number of pinch points and fragmentations.

## 5 CONCLUSION AND RECOMMENDATION

The abundance of Himalayan red panda is known to reduce in the areas accessible to livestock grazing due to its disturbances (Dendup et al., 2016; Sharma et al., 2014) and reduced bamboo growth to an optimum height preferred by a Himalayan red panda (Yonzon & Hunter, 1991). Livestock rearing is the main livelihood for the seminomadic inhabitants of SWS. Approximately, 75% of SWS is accessible to livestock with varying grazing intensity (SWS, 2019). Widespread herders and livestock are always accompanied by dogs which are known to carry canine distemper that is contagious to Himalayan red pandas through contact with feces and urine or a bite from infected dogs (Deem et al., 2000). The free‐roaming dog population is increasing in SWS due to the abandonment of old dogs by herders and their high birth rate. Incidences of dog hunting Himalayan red panda were reported in the studies elsewhere (Dorji et al., 2012; Yonzon & Hunter, 1991). Therefore, widespread of herders and increasing population of livestock and free‐roaming dogs could be severe threat to Himalayan red panda in SWS with more than 75% of the predicted potential habitats occurring outside the existing core zones.

Overall, SWS can play a critical role as a connecting link between the larger landscape of Bhutan and Arunachal Pradesh toward the conservation of Himalayan red panda that exhibits narrow dispersal with special habitat needs. Transboundary landscape connectivity will not only facilitate the genetic dispersal across geopolitical boundaries but also prepare for uninterrupted movement of Himalayan red panda and associated species in the event of habitat shift or expansion owing to future climate change (Rüter et al., 2014).

Though habitat and connectivity modeling tools used in current study possess some limitations, the findings suggest that existing Himalayan red panda habitats in SWS are unequally distributed across three ranges with a high frequency of anthropogenic disturbances. Yet, they can function as the important linkage in maintaining the transboundary movement of the Himalayan red panda in the larger landscape of Bhutan and Indian state of Arunachal Pradesh.

Taking into account the findings, following research actions are recommended:

1. To initiate GPS/satellite telemetry of the Himalayan red panda to understand their behavior and movement in the landscape to evaluate the functionality of the predicted habitat and least‐cost corridors. This can also help management of SWS in understanding the precise interaction between herders, large livestock, increasing population of free‐roaming dogs, and Himalayan red panda dwelling in the same landscape.
2. To explore the feasibility of transboundary conservation initiative with adjacent Indian state of Arunachal Pradesh to facilitate genetic dispersal of the species in a larger landscape. Such initiatives could also help in empowering the minor communities residing within and nearby the landscape, thus involving the communities toward conservation of the species.

## CONFLICT OF INTEREST

The authors have no conflict of interest to declare.

## AUTHOR CONTRIBUTIONS

**Sonam Tobgay:** Conceptualization (equal); Data curation (equal); Formal analysis (equal); Investigation (equal); Methodology (equal); Writing‐original draft (equal). **Nattapon Mahavik:** Conceptualization (equal); Supervision (equal); Validation (equal); Writing‐review & editing (equal).

## Supporting information


**Tables S1‐S4**

## Data Availability

Due to conservation threats, Himalayan red panda occurrence records used in this study will not be submitted to any public domain.
