# Supplementary material for: Potential habitat distribution of Himalayan red panda and their connectivity in Sakteng Wildlife Sanctuary, Bhutan
Source: Ecol Evol. 2020 Oct 28;10(23):12929–39. doi: 10.1002/ece3.6874 (PMC7713985; doi:10.1002/ece3.6874)
Supplement: Supplementary file 1 — Tables S1‐S4 [file ECE3-10-12929-s001.zip › Supplementary table 1 to 4/Supplementary information.docx]

**Supplementary information**

Table 1. Expert’s opinion on resistance cause by the different land use types. It ranges from 0 -1, where 0 is indicates least and 1 is the highest resistance

| **Land use** | **Experts rating** | | | | **Mean resistance** |
| --- | --- | --- | --- | --- | --- |
|  | **I** | **II** | **III** | **IV** |  |
| Alpine Scrubs | 0.8 | 0.1 | 0.5 | 0.6 | 0.5 |
| Broadleaf | 0.1 | 0.2 | 0.2 | 0.2 | 0.2 |
| Built up | 1.0 | 1.0 | 1.0 | 1.0 | 1.0 |
| Chirpine | 1.0 | 1.0 | 0.5 | 1.0 | 0.9 |
| Fir | 0.1 | 0.1 | 0.1 | 0.1 | 0.1 |
| Kamzhing (dry agricultural land) | 0.8 | 0.6 | 1.0 | 1.0 | 0.9 |
| Lake | 1.0 | 0.4 | 1.0 | 1.0 | 0.9 |
| Landslides | 1.0 | 0.4 | 1.0 | 1.0 | 0.9 |
| Meadows | 0.9 | 0.2 | 0.3 | 0.5 | 0.5 |
| Mixed conifer | 0.1 | 0.1 | 0.2 | 0.1 | 0.1 |
| Rivers | 1.0 | 0.8 | 1.0 | 1.0 | 1.0 |
| Rocky Outcrops | 1.0 | 0.1 | 0.5 | 1.0 | 0.7 |
| Shrubs | 0.7 | 0.3 | 0.5 | 0.5 | 0.5 |
| Snow and Glacier | 1.0 | 0.5 | 1.0 | 0.8 | 0.8 |
| Timber extraction sites (zone) | 0.3 | 0.2 | 0.4 | 0.5 | 0.4 |

Table 3 List of 19 bio-climatic and 3 environmental variables

| **Code** | **Description** |
| --- | --- |
| **Bio-climatic** | |
| BIO1 | Annual Mean Temperature |
| BIO2 | Mean Diurnal Range (Mean of monthly (max temp - min temp)) |
| BIO3 | Isothermality (BIO2/BIO7) (* 100) |
| BIO4 | Temp. Seasonality (standard deviation *100) |
| BIO5 | Max Temperature of Warmest Month |
| BIO6 | Min Temperature of Coldest Month |
| BIO7 | Temperature Annual Range (BIO5-BIO6) |
| BIO8 | Mean Temperature of Wettest Quarter |
| BIO9 | Mean Temperature of Driest Quarter |
| BIO10 | Mean Temperature of Warmest Quarter |
| BIO11 | Mean Temperature of Coldest Quarter |
| BIO12 | Annual Precipitation |
| BIO13 | Precipitation of Wettest Month |
| BIO14 | Precipitation of Driest Month |
| BIO15 | Precipitation Seasonality (Coefficient of Variation) |
| BIO16 | Precipitation of Wettest Quarter |
| BIO17 | Precipitation of Driest Quarter |
| BIO18 | Precipitation of Warmest Quarter |
| BIO19 | Precipitation of Coldest Quarter |
| **Environmental** | |
| Slope | Slope |
| Asp. | Aspect |
| Elev. | Elevation |

Table 4 The characteristics of 15 core habitats with their respective centrality scores and area corrected centrality scores.

| **Core habitats**  **(CH)** | **Area**  **(Km^2^)** | **Centrality scores**  **( amps)** | **Area corrected centrality scores (amps)** |
| --- | --- | --- | --- |
| 5 | 43.30 | 61.34 | 1.42 |
| 11 | 8.34 | 60.85 | 7.30 |
| 6 | 31.01 | 47.52 | 1.53 |
| 4 | 1.95 | 42.17 | 21.64 |
| 12 | 1.83 | 35.88 | 19.59 |
| 3 | 3.98 | 35.47 | 8.91 |
| 10 | 7.31 | 34.75 | 4.76 |
| 8 | 25.61 | 33.92 | 1.32 |
| 9 | 2.37 | 32.37 | 13.64 |
| 2 | 14.15 | 27.35 | 1.93 |
| 14 | 26.69 | 26.86 | 1.01 |
| 7 | 0.34 | 20.40 | 59.39 |
| 13 | 3.05 | 20.15 | 6.62 |
| 1 | 2.90 | 18.65 | 6.43 |
| 15 | 0.30 | 14.00 | 46.87 |
